# Multiple behavioural risk factors and mental health among adults in Estonia

**DOI:** 10.3389/fpubh.2025.1600598

**Published:** 2025-07-17

**Authors:** Galina Opikova, Rainer Reile, Kenn Konstabel, Kristjan Kask

**Affiliations:** ^1^Institute of Natural Sciences and Health, Tallinn University, Tallinn, Estonia; ^2^Department of Epidemiology and Biostatistics, National Institute for Health Development, Tallinn, Estonia; ^3^Department of Chronic Diseases, National Institute for Health Development, Tallinn, Estonia

**Keywords:** behavioural risk factors, lifestyle, latent class analysis, mental health, mental disorder (disease)

## Abstract

**Aim:**

Extensive evidence demonstrates the link between health behaviour and mental health. However, the impact of coinciding behavioural risk factors on mental health outcomes has received less attention. This study addresses this gap by analysing multiple behavioural risk factors and their association with mental health.

**Subject and methods:**

Nationally representative data (*n* = 6,404) from 2020 cross-sectional survey in Estonia was used to examine patterns of co-occurring behavioural risk factors, including smoking, alcohol consumption, physical inactivity, unhealthy diet, drug use, and high screen time. Latent class analysis (LCA) was employed to identify behavioural classes, and binomial logistic regression was used to examine associations between predicted individual class membership and self-reported mental health outcomes, such as depressiveness, stress, suicidal thoughts, diagnoses of depression and insomnia, and medication use.

**Results:**

LCA identified three behavioural classes: multiple risk factors (14.6%), low-risk lifestyle (79.9%), and drug use lifestyle (5.5%). Compared to individuals in the low-risk lifestyle class, respondents in the multiple risk factors and drug use classes had higher odds of experiencing depressiveness, stress, and suicidal thoughts, as well as self-reported diagnoses of depression and insomnia; they also exhibited increased use of medications, such as antidepressants, hypnotics, and sedatives.

**Conclusion:**

Behavioural risk classes were associated with adverse mental health outcomes. These findings emphasise the importance of focused interventions targeting these risk factors to address the risk of mental health problems.

## Introduction

Behavioural risk factors, such as low physical activity and sedentary behaviour, unhealthy diet, and alcohol, tobacco, and drug use, contribute to the aetiology of many non-communicable diseases (1) and increase the risk of mental health problems (2). In 2017, behavioural risk factors contributed to 23.8 million deaths and 913 million lost disability-adjusted life years (DALYs) (3). Specifically, in the context of mental health, these risks were associated with the loss of 8 million DALYs. Since exposure to behavioural risks during adulthood contributes to the majority of the disease burden (1), focusing on the patterning of these risks at this life stage is vital for enhancing knowledge on the behavioural determinants of health. Furthermore, *g*iven the high societal cost of mental health disorders (4), prioritising efforts to reduce the disease burden related to preventable behavioural risks is crucial.

Previous studies have established that behavioural risk factors are interrelated and often co-occur (5, 6), leading to potentially poorer health outcomes (7, 8). As co-occurrence-based methods can help to identify sub-groups for targeted interventions (9), understanding the association between different patterns of behavioural risk and mental outcomes is essential for developing appropriate and effective interventions. A growing body of literature examines lifestyle patterns and their links to mental health across European countries (10–16). Most of these studies (10, 11, 15, 16) have focused on anxiety or depression, examining their association with modifiable health behaviours such as smoking, alcohol consumption, physical inactivity, and unhealthy diet. Findings indicate that unhealthier behavioural patterns increase the risk of depression, drug and alcohol dependence, and social phobia (10, 16) and also result in higher levels of psychological distress (11).

This study focuses on Estonia, where previous research has confirmed the link between mental health outcomes and behavioural risk factors (7, 17, 18). However, these studies did not consider the co-occurrence of behavioural risks. A recent study (7) indicated that exposure to three or more behavioural risk factors contributes to higher odds of mental health problems. Considering the combined effect of multiple risk behaviours and the potential benefits of targeted health interventions addressing co-occurring risks (19), it is important to investigate multiple behavioural risk factors and their connection to mental health outcomes.

The overall aim of the study is to analyse the associations between multiple behavioural risk factors and mental health among adults in Estonia. More specifically, the study will: (a) explore the patterns of co-occurring behavioural risk factors and (b) analyse their association with mental health outcomes.

## Methods

### General study design

This study utilized data from the Estonian cross-sectional survey Health Behaviour among Estonian Adult Population conducted in 2020. A nationally and regionally representative sample (stratified by sex, five-year age groups and 17 regional strata) of 12,400 individuals aged 16–64 years as of 1 January 2020 was drawn from population register. Data were collected using combined postal and web survey between March and June 2020. In total, 6,404 valid responses were obtained with a crude response rate being 51.6%. The study was approved by the Tallinn Medical Research Ethics Committee (approval no. 2839 and 2,840, 26.06.2019). Detailed information about the survey is available elsewhere (20).

### Health behaviour variables

Drawing on earlier studies, we identified key health behaviour indicators, including smoking, alcohol consumption, physical inactivity, unhealthy diet, drug use, and high screen time. The daily smoking variable was binary (yes, no) based on the item “Have you ever smoked in your life? “Alcohol consumption (high risk, low risk) was calculated based on self-reported consumption of alcohol and measured in alcohol units (10 g of pure alcohol) over the past 7 days with high risk referring to consumption of ≥14 alcohol units for men and ≥7 units for women. Drug use was assessed using a single-item question, “Have you used or tried narcotic substances or prescription medicines without doctors’ prescription?” and use within the past 12 months was classified as a risk behaviour. Based on World Health Organisation (WHO) (21) recommendation that free sugar intake should be less than 10% of total energy intake, an unhealthy diet was defined as the consumption of sugar-rich products (candies, chocolate, cakes, biscuits, sweet pastries, juice, flavoured water, energy drinks) on ≥6 days in the past week. This definition was based on a predefined list of items included in the questionnaire. To assess physical activity, participants were asked “How often in your leisure time do you exercise for at least half an hour so that you will breathe a bit heavier and sweat a little? “Response options were dichotomised into inactive (physical exercise less than once a week), active (physical exercise once a week or more frequently). Screen time, defined as sedentary behaviour, referred to self-reported average time spent on electronic devices (e.g., TV, computer, smartphone) during leisure time over the past 30 days. According to the WHO guidelines (22), adults should limit the time spent in sedentary activities, including screen time. Therefore, a daily screen time of ≥6 h was considered a behavioural risk factor.

### Mental health variables

Six self-reported mental health outcomes were included in the study, divided into mental health complaints (depressiveness, stress, and suicidal thoughts) and mental health related diagnoses (depression, insomnia) or medication use. Depressiveness was evaluated within the item “In the past 30 days, have you been unhappy, depressed?” with response dichotomised into yes (“yes, a lot more than before,” “yes, somewhat more than before”) or no (“yes, but no more than before,” “not at all”). Perceived stress was assessed with the item “In the past 30 days, have you been stressed, under pressure? “with response options grouped into yes (“yes, my life is almost unbearable,” “yes, more than people on the average”) or no (“yes, but no more than people on the average,” “not at all”). Suicidal thoughts were assessed with the item “Have you ever thought about suicide?” and categorised into yes (“yes, during the past 12 months,” “yes during the past 12 months and earlier”) or no (“no,” “yes, earlier”). Binary variables (yes, no) on self-reported diagnosis or treatment for depression in the past 12 months, and sel-reported insomnia complaints during the past 30 days were also included. Medication intake was assessed with the question, “In the past 7 days, have you taken any medications or supplements?” Responses indicating the use of antidepressants, hypnotics, or sedatives were classified as mental health-related medication use.

### Statistical analysis

Descriptive statistics were used to characterise the data based on the proportions of mental health complaints and self-reported diagnoses. Differences between groups were tested using chi-square test and post-hoc test with Bonferroni correction used for multiple comparisons.

Latent class analysis (LCA), applied to six behavioural indicators defined beforehand, was used to study the patterns of multiple behavioural risk factors. LCA is a probabilistic, unsupervised and person-centred clustering method that detects distinct subgroups that share common characteristics, primarily relying on maximum likelihood estimation (23). The analysis included testing different models with up to four class solutions (Supplementary Table 1). To determine the best-fitting model, Bayesian information criteria (BIC), Akaike information criterion (AIC) (23) and entropy were compared and ranked. Based on the low AIC (29919) and BIC (30053) values and the highest entropy (0.541), a three-class model was selected. Although the entropy value indicates moderate classification certainty, it was considered acceptable in combination with theoretical justification and the interpretability of the classes.

The associations between individual behavioural risk classes and mental health outcomes were examined using binomial logistic regression. Predicted individual class membership values were treated as independent variables, with class 2 (low-risk lifestyle) used as the reference category. Univariate and adjusted (by sex and age) models were run separately for each mental health indicator. The results of binomial logistic regression were presented as odds ratios (OR) with 95% confidence intervals (CI). All analyses were performed using Jamovi software version 2.3.28, based on R packages (24).

## Results

The key characteristics of the data by sex, age, and behavioural risk factors are presented in Table 1. With respect to mental health outcomes, more than half (54.4%) of respondents reported at least one mental health problem, but considerable variation was found across demographic and health behaviour variables. Females reported significantly more mental health problems compared to males for all indicators considered. A distinct age gradient was observed for mental health complaints, with symptoms reported more frequently among younger respondents. All mental health outcomes were significantly more common among individuals with low physical activity or drug use. Furthermore, all mental health outcomes (except medication use) varied significantly by smoking status and screen time. Respondents with an unhealthy diet generally showed a higher proportion of mental health outcomes, except for insomnia, where difference was non-significant. In contrast, alcohol consumption showed statistically significant variation only for stress and suicidal thoughts. All mental health outcomes were significantly more common among individuals in the multiple risk factors and drug use lifestyle classes, compared to low-risk class.

**Table 1:** Characteristics of participants by proportions of mental health outcomes (*n* = 6,040).

Variables	*n* (%)	Mental health complaints (%)			Self-reported diagnoses/medication use (%)		
		Depressiveness	Stress	Suicidal thoughts	Depression	Insomnia	Medication use
Social-demographic factors							
Sex							
Female	3,467 (57.4%)	24.7%^c^	22.9%^c^	19.7%^c^	13.2%^c^	40.5%^c^	19.4%^c^
Male	2,573 (42.6%)	17.5%^c^	18.5%^c^	16.4%^c^	8.7%^c^	32.1%^c^	12.2%^c^
*Total*	6,040 (100%)	21.6%	21.0%	18.3%	11.3%	36.9%	16.3%
Age							
16–24	817 (13.5%)	30.0%^c^	28.3%^c^	31.6%^c^	11.3%	40.4%^b^	11.8%^c^
25–34	1,160 (19.2%)	21.7%	23.2%^a^	20.0%^c,a^	7.9%^b,c^	32.2%^b,c^	10.0%^c^
35–44	1,145 (19.0%)	20.6%	19.8%^c^	18.2%^b^	10.4%	32.4%^b,c^	13.1%^b^
45–54	1,408 (23.3%)	19.6%	18.9%^c^	15.1%^c,a^	12.3%^b^	36.9%	18.5%^b,c^
55–64	1,510 (25.0%)	19.8%	18.5%^c,a^	12.8%^c,b^	13.6%^c^	42.1%^c^	24.1%^b,c^
Behavioural risk factors							
Risky drinking							
Yes	911 (15.7%)	23.5%	23.5%^a^	24.3%^c^	11.9%	39.9%	15.9%
No	4,898 (84.3%)	21.0%	20.3%^a^	17.1%^c^	11.1%	36.5%	16.3%
Low physical activity							
Yes	2,293 (40.7%)	24.4%^c^	23.2%^c^	20.1%^b^	13.1%^c^	40.3%^c^	19.7%^c^
No	3,344 (59.3%)	19.7%^c^	19.4%^c^	16.9%^b^	9.9%^c^	34.9%^c^	14.2%^c^
Daily smoking							
Yes	1,122 (18.9%)	25.5%^c^	23.7%^b^	22.1%^c^	13.6%^b^	42.0%^c^	17.8%
No	4,828 (81.1%)	20.6%^c^	20.3%^b^	17.4%^c^	10.8%^b^	35.9%^c^	16.0%
Drug use							
Yes	381 (6.5%)	31.1%^c^	34.5%^c^	39.4%^c^	15.5%^b^	49.7%^c^	23.6%^c^
No	5,473 (93.5%)	20.8%^c^	19.8%^c^	16.8%^c^	11.0%^b^	36.2%^c^	15.7%^c^
Unhealthy diet							
Yes	877 (16.1%)	24.6%^b^	23.9%^a^	23.2%^c^	13.6%^b^	39.6%	18.6%^b^
No	4,576 (83.9%)	20.6%^b^	20.2%^a^	17.2%^c^	10.3%^b^	36.4%	15.7%^b^
High screen time							
Yes	748 (13.7%)	29.0%^c^	27.6%^c^	27.0%^c^	13.5%^b^	42.5%^c^	17.8%
No	4,693 (86.3%)	19.8%^c^	19.4%^c^	16.5%^c^	10.4%^b^	35.7%^c^	15.7%
Behavioural classes							
Low-risk lifestyle	4,828 (79.9%)	20.2%^c^	19.8%^c,a^	16.2%^c^	10.7%^a^	35.2%^c^	15.7%^a^
Multiple risk factors	880 (14.6%)	27.0%^c^	23.7%^a^	22.5%^c^	13.8%^a^	42.2%^c^	17.8%
Drug use lifestyle	332 (5.5%)	28.7%^c^	32.6%^c^	37.3%^c^	13.3%	47.4%^c^	21.4%^a^

Post-hoc test and chi-square test indicate statistical differences between column proportions by predictor variables (a = *p* < 0.05, b = *p* < 0.01, c = *p* < 0.001).

**Figure 1:**
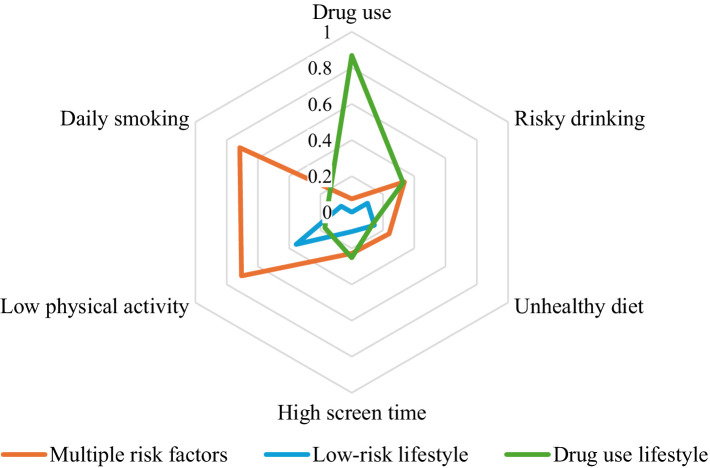
Estimated class-specific response probabilities for six behavioural risk factors.

Figure 1 illustrates the response probabilities for six behavioural risk factors across the three latent classes identified through LCA. Class 1 (*n* = 880, 14.6% of respondents) is labelled “multiple risk factors” and is mostly defined by a higher probability of daily smoking, low physical activity, and high alcohol consumption. Class 2 (*n* = 4,828, 79.9% of respondents) is characterized as the “low-risk lifestyle” class, defined by generally low probability for most of the behavioural risk factors considered (except for moderate probability of low physical activity). Class 3 (*n* = 332, 5.5% of respondents) labelled as “drug use lifestyle” in this study, is characterized by the highest probability of drug use, compared to other classes and risky drinking at levels similar to Class 1.

Regression analysis (Table 2) revealed strong associations between behavioural classes and various mental health outcomes. Respondents in the multiple risk factors class had higher odds for experiencing depressiveness (OR 1.72; CI 1.45–2.03), stress (OR 1.40; CI 1.18–1.67) and suicidal thoughts (OR 1.74; CI 1.45–2.09) compared to the low-risk lifestyle class even after adjusting for sex and age. A similar pattern was observed also for self-reported diagnoses and medication use with respondents in multiple risk factors class (compared to low-risk lifestyle class) having higher odds for being diagnosed or treated for depression, experiencing insomnia or using mental health-related medications.

**Table 2:** Results of the binomial logistic regression models (OR and 95% CI) for associations between behavioural risk classes and mental health outcomes.

Mental health outcomes	Multiple risk factors vs. Low-risk lifestyle		Drug use lifestyle vs. Low-risk lifestyle	
	OR (95% CI)		OR (95% CI)	
	Model 1	Model 2	Model 1	Model 2
Mental health complaints				
Depressiveness	1.46 (1.24–1.72)^c^	1.72 (1.45–2.03)^c^	1.59 (1.24–1.04)^c^	1.50 (1.15–1.95)^b^
Stress	1.26 (1.06–1.49)^b^	1.40 (1.18–1.67)^c^	1.97 (1.55–2.50)^c^	1.79 (1.39–2.31)^c^
Suicidal thoughts	1.50 (1.26–1.80)^c^	1.74 (1.45–2.09)^c^	3.09 (2.44–3.91)^c^	2.46 (1.92–3.16)^c^
Self-reported diagnoses/medication use				
Depression	1.33 (1.08–1.65)^b^	1.49 (1.20–1.86)^c^	1.28 (0.92–1.78)	1.65 (1.17–2.34)^b^
Insomnia	1.34 (1.16–1.55)^c^	1.48 (1.27–1.72)^c^	1.66 (1.33–2.08)^c^	1.95 (1.54–2.47)^c^
Medication use	1.17 (0.96–1.41)	1.29 (1.06–1.57)^a^	1.46 (1.11–1.92)^b^	2.58 (1.91–3.47)^c^

a = *p* < 0.05, b = *p* < 0.01, c = *p* < 0.001.

Model 1 – unadjusted.

Model 2 – adjusted for sociodemographic factors (sex, age).

In the unadjusted models, respondents in the drug use lifestyle class had significantly higher odds of all mental health outcomes except depression, compared to the low risk lifestyle class. After adjusting for sex and age, the association was slightly attenuated for mental health complaints but increased for items in self-reported diagnoses and medication use. In the adjusted model, respondents in the drug use lifestyle had 1.5–2.5 times higher odds of all mental health items compared to the low-risk lifestyle class, with the largest difference found for suicidal thoughts (OR 2.46; CI 1.92–3.16) and medication use (OR 2.58; CI 1.91–3.47).

## Discussion

In this study, we explored the patterns of co-occurring behavioural risk factors and their association with various mental health outcomes. Based on six individual health behaviour indicators, three distinct behavioural classes were identified: multiple risk factors, low-risk lifestyle, and drug use lifestyle. Compared to the low-risk lifestyle class, respondents in the multiple risk factors or the drug use lifestyle classes had substantially higher odds of all mental health outcomes considered in the study.

These findings align with earlier research suggesting that interrelated and often coinciding (5, 6) behavioural risk factors contribute to poorer mental health outcomes (7, 8). Prior studies using the same statistical techniques have found that individuals within unhealthy behavioural classes are more likely to report symptoms of anxiety, depression, and stress (10, 16, 25). In line with this, our study revealed that individuals in the multiple risk factors and drug use lifestyle classes had higher odds of experiencing depressiveness, stress, and being diagnosed with depression.

Consistent with previous studies from Estonia (7, 26), we also found a strong association between drug use and mental health outcomes. However, direct comparisons to these studies are challenging due to differing analytical approaches. Nevertheless, our findings support the broader literature linking suicidal thoughts to combined behavioural risk factors such as problematic alcohol use, drug use, and smoking (27–29). Individuals in the drug use lifestyle class showed the highest odds of suicidal thoughts. This may be attributed to the fact that they often experience social isolation, economic hardship, and stigma, all of which are known to increase the risk of suicidal behaviours (29).

Similar to previous studies (30, 31), we found an association between insomnia and both the multiple risk factors and drug use classes. This relationship may be bidirectional – on the one hand, alcohol use results in poorer sleep quality (32), while on the other, it is possible that individuals with insomnia may use alcohol or drugs as a remedy for their sleep problems (30, 33). Importantly, the finding that individuals in the drug use class had the highest probability of medication use is alarming, as guidelines for medication use strictly advise against combining medications with alcohol or drugs (34).

From the identified three behavioural classes, 79.9% respondents were classified to low-risk lifestyle class, which is consistent with previous studies, where more than three-quarters of the sample belonged to the healthier group (16, 25). Although national-level evidence on behavioural clustering is limited due to predominant focus on individual behaviour indicators, a recent study (7) using the same data found that a quarter of Estonian adults aged 16 to 64 were not exposed to any health behavioural risk factors, while one in five was exposed to three or more – a pattern, which aligns with our results from LCA model, where 14.6% of respondents were classified into multiple risk factors class. These finding align with systematic reviews suggesting that smoking and risky alcohol use are more likely to co-occur (19). Moreover, our results showed coincidence between drug use and smoking in the smallest class – drug use lifestyle (5.5%). In the context of class proportions, the results of previous studies regarding unhealthy behavioural classes depended on different factors, including sample size and variety of variables, and ranged from less than 2 to 40% (10, 16, 19, 25).

Prior studies have demonstrated that socio-demographic factors additionally differentiate the association between multiple behavioural risk factors and mental health (10, 11, 16). Although demographic indicators were primarily used to adjust for potential confounding between lifestyle and mental health outcomes in regression models, their variation across mental health indicators is noteworthy (7). In our analysis, we observed an indirect impact on the association between behavioural classes and mental health outcomes. While this impact varied across different variables, we found that the effect of behavioural classes differed by age, particularly in relation to indicators of depressiveness, insomnia, and medication use.

Additionally, divergent findings have been reported regarding the prevalence of mental health outcomes across gender. While some studies indicate elevated rates among males (25, 31), while other studies identify a higher proportion among females (35, 36). Our study results align with the latter, as we observed higher proportions of all mental health outcomes among females compared to males. A significantly high proportion of mental health outcomes, such as depressiveness, stress, and suicidal thoughts, were found in younger age groups – particularly among those aged 16–24. These results are consistent with those of previous studies (35). However, self-reported depression diagnoses and medication use were higher in the 55–64 age group. One possible explanation is that younger individuals face greater obstacles in help-seeking, including stigma, embarrassment, and a preference for self-reliance (37), reinforcing the need for preventative strategies among youth. In addition, further studies incorporating a wider set of socio-economic variables could potentially provide additional insights into these disparities.

When interpreting these findings, several aspects regarding the data and methods used should be acknowledged. First, the cross-sectional nature of the data does not allow to determine causality between behavioural risk classes and mental health outcomes. Thus, the results showing a strong association between behavioural risk classes and mental health outcomes should be interpreted as correlational, and further longitudinal research is warranted to establish causality. Second, the survey data relies on self-reported indicators of both health behaviour and mental health, which may introduce recall bias and social desirability bias, and cannot be externally validated. In addition, the operationalization of some behavioural risk factors was limited by the structure of the questionnaire, meaning that the use of specific cut-off values may not be directly comparable to those used in validated instruments. However, the study is based on a repeated cross-sectional survey with the core questionnaire and methods being consistent since the 1990s, with the long-term trend-data suggesting good concurrent validity (20). Furthermore, the inclusion of six differently conceptualised mental health items, for which behavioural risk factors retained their significance, provides confidence in the overall findings. Third, lifestyle patterns are defined using the LCA model that assigns respondents to classes, but the observed health behaviour patterns across the six variables may not always match the estimated class membership. Additionally, the entropy value of the selected model suggests moderate class separation, indicating some uncertainty in class assignment. However, we performed an additional analysis (Supplementary Table 2), where a group characterized by daily smoking and physical inactivity and another group based on drug use, yielded similar results in the regression analysis for mental health outcomes compared to the low-risk group.

Despite these limitations, the strengths of the study include a large nationally and regionally representative sample and the use of LCA methods. Furthermore, the study addresses a notable gap by examining the association between multiple behavioural risk factors and mental health among adults in Eastern Europe, providing a new perspective on understanding the co-occurrence of behavioural risk factors and their link with mental health outcomes.

## Conclusion

This study examined the association between multiple behavioural risk factors and mental health among the adult population. Our findings indicate that individuals with co-occurring behavioural risk factors have poorer mental health outcomes compared to those with a low-risk lifestyle. These findings contribute to the broader understanding that addressing multiple risk behaviours is essential in preventing negative health impacts.

## Data Availability

The data analyzed in this study is subject to the following licenses/restrictions: the dataset used in this study is not publicly available due to data protection regulations and participant confidentiality. Access to the data may be granted upon reasonable request and with permission from the data owner. Requests to access these datasets should be directed to galina.opikova@tlu.ee.
