# Supplementary material for: Multiple behavioural risk factors and mental health among adults in Estonia
Source: Front Public Health. 2025 Jul 17;13:1600598. doi: 10.3389/fpubh.2025.1600598 (PMC12311631; doi:10.3389/fpubh.2025.1600598)
Supplement: Supplementary file 1 [file Table_1.docx]

***Supplementary material***

**Table 1.** Goodness-of-fit measures of the four investigated models for deciding the number of classes.

| **Number of classes** | **AIC** | **BIC** | **ABIC** | **Entropy** |
| --- | --- | --- | --- | --- |
| Class 1 | 30422 | 30463 | 30444 | - |
| Class 2 | 30053 | 30140 | 30099 | 0.405 |
| Class 3 | 29919 | 30053 | 29989 | 0.541 |
| Class 4 | 29875 | 30056 | 29970 | 0.417 |

**Table 2.** Results of the binomial logistic regression models (OR and 95% CI) for associations between drug use group and multiple risk behaviour group (daily smokers and physically inactive respondents) and mental health outcomes.

| **Mental health outcomes** | **Multiple risk behaviours *vs* 0 risk behaviours**  **OR (95% CI)** | **Drug users *vs* Non-drug users**  **OR (95% CI)** |
| --- | --- | --- |
| *Mental health complaints* |  |  |
| Depressiveness | 1.59 (1.30–1.94)^c^ | 1.55 (1.21–1.98)^c^ |
| Stress | 1.40 (1.14–1.71)^c^ | 1.85 (1.45–2.35)^c^ |
| Suicidal thoughts | 1.62 (1.31–2.00)^c^ | 2.45 (1.93–3.11)^c^ |
| *Self-reported diagnoses and medications* | | |
| Depression | 1.31 (1.01–1.70)^a^ | 1.85 (1.34–2.56)^c^ |
| Insomnia | 1.49 (1.25–1.77)^c^ | 2.03 (1.62–2.54)^c^ |
| Medication use | 1.30 (1.04–1.63)^a^ | 2.83 (2.13–3.74)^c^ |
| Note. a = p<0.05, b = p<0.01, c = p<0.001.  Adjusted for sociodemographic factors (sex, age) | | |
